# Die Flexion zur Entlassung ist kein Prädiktor der Gelenkfunktion ein Jahr nach Knietotalendoprothese

**DOI:** 10.1007/s00132-022-04327-5

**Published:** 2022-11-30

**Authors:** Janice Richter, Georg Matziolis, Uwe Kahl

**Affiliations:** 1 Deutsches Zentrum für Orthopädie Waldkliniken Eisenberg, Universitätsklinikum Jena, Klosterlausnitzer Str. 81, 07607 Eisenberg, Deutschland; 2 Orthopädisches Zentrum, Sportklinik Erfurt, Erfurt, Deutschland Am Urbicher Kreuz 7, 99099

**Keywords:** Totale Knieendoprothese (Knie-TEP), Outcome, Range of Motion (ROM), Knee Society Score (KSS), Fast-Track Surgery, Total knee arthroplasty (TKA), Treatment outcome, Range of motion (ROM), Knee Society Score (KSS), Enhanced recovery after surgery

## Abstract

**Thema:**

In der Knieendoprothetik spielt das Bewegungsausmaß des operierten Gelenks eine wichtige Rolle. Als Qualitätskriterium wird eine Flexion von mindestens 90° zum Entlassungszeitpunkt angesetzt und als prädiktiver Wert für das Outcome ein Jahr nach Operation unterstellt. Dieser Zusammenhang ist dabei allerdings noch nicht belegt und soll in der vorliegenden Arbeit thematisiert werden.

**Methodik:**

Insgesamt wurden die Daten von 182 Patienten bzw. Gelenken retrospektiv ausgewertet. Outcomes wurden präoperativ, zur Entlassung, nach 6 Wochen und nach einem Jahr postoperativ erfasst. Zur Beantwortung der Fragestellung wurde das Bewegungsausmaß (ROM) des Kniegelenks ermittelt sowie KSS, SF-36, WOMAC, EQ-5D und VAS zur Beurteilung von Funktion und Lebensqualität erhoben. Es wurden 2 Gruppen abhängig vom Erreichen des 90°-Ziels zur Entlassung erstellt und nach 6 Wochen sowie ein Jahr nach Operation miteinander verglichen.

**Ergebnisse:**

Die Flexion des Kniegelenks zwischen den beiden Gruppen war zum Entlassungszeitpunkt (E) mit 91° gegenüber 70° signifikant unterschiedlich (*p* < 0,001). Nach 6 Wochen näherten sich die Flexionswerte auf 112° ± 13° (E > 90°) vs. 106° ± 14° (E < 90°) an (*p* = 0,001). Ein Jahr postoperativ konnte bei einer Flexion von durchschnittlich 122° ± 10° (E > 90°) vs. 120° ± 10° (E < 90°) weder ein Unterschied bezüglich der ROM (*p* = 0,57) noch bezüglich der Funktion oder Lebensqualität in sämtlichen erhobenen Scores zwischen den beiden Gruppen festgestellt werden.

**Schlussfolgerung:**

Nach den Ergebnissen dieser Studie ist das 90°-Kriterium kein adäquater Indikator für die mittelfristige Ergebnisqualität nach Knietotalendoprothese. Es kann weder ein Vorteil noch ein Nachteil durch das Erreichen einer 90°-Flexion zum Entlassungszeitpunkt festgestellt werden.

## Abstract (en)

**Objective:**

In total knee arthroplasty (TKA), range of motion has become established as an important factor. The criterion of quality is set to flexion of 90° after hospitalisation. Currently, it is supposed to be a predictor for 1‑year postoperative outcome. However, as this correlation has not been proven, this clinical trial was performed.

**Methods:**

A total of 182 TKA recipients were analysed retrospectively. Outcomes were assessed before surgery, after hospitalization, 6 weeks and 1 year post surgery. They included knee range of motion (ROM) to answer the main hypothesis, but also KSS, SF-36, WOMAC, EQ-5D and VAS to evaluate knee function and quality of life. The patients were divided into two groups differing in achieving 90° flexion after hospitalisation and compared 6 weeks and 1 year after surgery.

**Results:**

Knee flexion differed significantly between groups from 91 to 70° in the group without the aim of 90° flexion after hospitalisation (E) (*p* < 0.001). After 6 weeks, flexion was improved to 112° ± 13° (E > 90°) vs. 106° ± 14° (E < 90°; *p* = 0.001). One year post surgery, knee flexion averaged 122° ± 10° (E > 90°) vs. 120° ± 10° (E < 90°) with no difference between the groups (*p* = 0.57) and no significant difference in all other scores concerning knee function and quality of life.

**Conclusion:**

Flexion of 90° after hospitalisation is not adequate to predict medium-term outcomes after TKA. There is no advantage or disadvantage regarding whether the aim of 90° flexion is achieved.

## Einleitung

In Deutschland werden jährlich über 200.000 künstliche Kniegelenke implantiert [10]. Ein etabliertes Entlassungskriterium des Instituts für Qualitätssicherung und Transparenz im Gesundheitswesen (IQTiG) ist die Flexionsfähigkeit des operierten Kniegelenks zum Entlassungszeitpunkt. Diese soll mindestens 90° betragen [10]. Grundlage für das Qualitätskriterium „Beweglichkeit bei Entlassung“ bilden verschiedene Studien und Berichte bis 2012 [9, 15, 18]. Dabei wird unterstellt, dass dieses Entlassungskriterium ein Ergebnisqualitätskriterium ist. Ein solches sollte prädiktiv für die längerfristige, mindestens jedoch mittelfristige Funktion des Kniegelenks sein. Ein solcher Zusammenhang ist trotz langjähriger Anwendung dieses Qualitätskriteriums bislang nicht belegt.

Optimierungsprozesse gewinnen im Gesundheitssystem gerade bei elektiven Eingriffen aufgrund der begrenzten Ressourcen zunehmend an Bedeutung. Im Fokus steht dabei unter anderem die Verkürzung der Hospitalisierungszeit, sofern die Compliance, das Verantwortungsbewusstsein und die Mitarbeit des Patienten dies zulassen [4, 13, 24]. Jenes Vorgehen wird unter anderem als Fast-Track Surgery bezeichnet und ermöglicht neben ökonomischen Einsparungen durch verringerte Betten- und Personalbindung auch die Reduktion allgemeiner Komplikationen [1, 2, 14, 22]. Auch in Deutschland hat sich seit Einführung des DRG-Systems die Krankenhausverweildauer nach Knie-TEP von 17 Tagen im Jahr 2003 auf nur noch 8,6 Tage im Jahr 2019 reduziert [10]. In Dänemark erfolgt sogar eine Entlassung am 2. postoperativen Tag (POD) [13].

Trotz moderner Operationsverfahren und optimiertem perioperativen Management ist in vielen Fällen eine Flexion von 90° zu einem so frühen Zeitpunkt nicht zu erreichen. Es ist bislang unklar, ob dies für die betroffenen Patienten einen Nachteil darstellt und die frühe Flexionsfähigkeit einen prädiktiven Wert für die Funktion des operierten Gelenks ein Jahr nach der Operation hat. Dies impliziert den Sinn einer 90°-Flexion als Entlassungskriterium nach Knietotalendoprothese.

## Methodik

Bei dieser Studie handelt es sich um eine retrospektive Auswertung von Patientendaten, die im Rahmen einer prospektiven Studie erhoben wurden. Der Studienzeitraum lag zwischen August 2018 und August 2020. Diese wurde von der hiesigen Ethikkommission genehmigt (5541-05/18). Für die Bestimmung einer minimalen Patientenzahl wurde im Vorfeld eine Power-Analyse (G-Power) durchgeführt. Als primäres Kriterium des Behandlungserfolgs nach Knie-TEP wurde das Bewegungsausmaß des Patienten zum Endzeitpunkt festgelegt. Zielgröße war dabei eine Flexion von 125°, wobei ab einer Verschlechterung um 5° auf 120° von einem klinisch relevanten Effekt ausgegangen werden kann. Als Standardabweichung wurden 10 % Abweichung vom Mittelwert angenommen, also 12°. Damit ergab sich für ein Signifikanzniveau von 0,05 und eine Power von 0,80 die notwendige Fallzahl von 72 Patienten pro Gruppe. Diese Größenordnung entspricht auch der Fallzahl vergleichbarer Studien aus der Literatur [18, 26].

## Ein- und Ausschlusskriterien der Studie

Insgesamt wurden 182 Patienten bzw. 182 Gelenke nach den folgenden Kriterien in die Studie eingeschlossen (Tab. 1). Aus den Daten der zugrunde liegenden prospektiven Studie wurden folgende etablierte Parameter und Scores verwendet [16, 23]: Alter, Geschlecht, ASA-Score, EQ-5D-5L, Knie-Society-Score (KSS), visuelle Analogskala für Schmerzen (VAS), SF-36 und Western Ontario and McMaster Universities Osteoarthritis Index (WOMAC). Diese wurden präoperativ, nach 6 Wochen und ein Jahr postoperativ erfasst. Zum Entlassungszeitpunkt wurde das Bewegungsausmaß des operierten Gelenks dokumentiert.

Bei der endoprothetischen Versorgung wurde für alle Patienten ein standardisierter Ablauf angewandt: behandelnder Arzt/Operateur, Anästhesie- und Operationstechnik sowie intra- und postoperative Schmerztherapie waren identisch. Es kamen Prothesensysteme der Firma Smith & Nephew®, London, Vereinigtes Königreich zum Einsatz – das LEGION-Primär-System oder alternativ das GENESIS-II-System sowie das Journey-BCS-II-System. Präoperativ erfolgten eine orale Prämedikation mit Benzodiazepinen und eine gewichtsadaptierte Antikoagulation mit niedermolekularem Heparin. Die Narkose wurde als totalintravenöse Anästhesie mit Larynxmaske, proximalem Femoralis- und Ischiadikusblock sowie lokaler Infiltrationsanästhesie durchgeführt. Es wurden zudem ein Antifibrinolytikum, Prednisolon und eine Antibiose mittels Cephalosporinen verabreicht. Die Operationstechnik entsprach einer Femur-first-Technik mit mechanischem Alignment und Erhalt des hinteren Kreuzbands und war bei allen Patienten gleich.

Die postoperative Schmerztherapie erfolgte in beiden Gruppen nach einem Standardprotokoll entsprechend dem WHO-Stufenschema. Die innerklinische Frührehabilitation erfolgte ab dem Morgen des 1. POD durch das Pflegepersonal sowie ab dem 2. POD zusätzlich am Nachmittag durch die Physiotherapie. Die passive Mobilisierung auf der Bewegungsschiene begann am 2. POD bei 40° Flexion mit einer Steigerung von 5–10° pro Tag. Das Prozedere setzte sich bis zur Entlassung fort, wobei die Mobilisation individuell ausgeweitet wurde.

Die Entlassungskriterien inkludierten explizit nicht die Flexionsfähigkeit zum Zeitpunkt der geplanten Entlassung, sondern orientierten sich an den Kriterien von vorhergehenden Studien zu Fast-Track Surgery aus Dänemark [13]. Der Patient musste sich zum Zeitpunkt der Entlassung ohne Hilfe Ankleiden können, die persönliche Hygiene ausführen, aus dem Bett ein- und aussteigen und sich selbstständig auf einen Stuhl bzw. die Toilette setzen und wieder aufstehen können. Bei Mobilisation mit Unterarmgehstützen musste der Patient eine Distanz von 70 Metern zurücklegen können sowie einen Treppenabsatz bewältigen. Zudem sollte die Schmerzangabe auf der VAS unter Medikation kleiner als 5 sein. Mit diesen Entlassungskriterien ergab sich eine individuelle Krankenhausverweildauer zwischen 3 und 10 Tagen.

Nach Entlassung erfolgte nach Patientenwunsch eine stationäre oder ambulante Anschlussheilbehandlung (AHB) nach standardisiertem Rehabilitationsprotokoll. Eine Überlegenheit einer stationären oder ambulanten Durchführungsform ist bisher nicht belegt, sodass dieser Parameter nicht als Confounder erfasst und gewertet wurde [17].

Für die Beantwortung der Fragestellung wurden die Patienten nach ihrer ROM zum Entlassungszeitpunkt in 2 Gruppen unterteilt (weniger und mehr als 90° Flexion). Auf Gruppenunterschiede bezüglich aller erfassten Parameter wurde mittels t‑Test für unverbundene Stichproben auf einem Signifikanzniveau von *p* = 0,05 getestet.

## Ergebnisse

Entsprechend der Ein- und Ausschlusskriterien wurden 182 Patienten in die Studie eingeschlossen (Tab. 1). Das Patientenalter wurde als Differenz zwischen Op.-Datum und Geburtsdatum definiert und lag geschlechtsunabhängig im Durchschnitt bei 65,3 ± 8,3 (38,6–87,5) Jahren. In beiden lag der ASA-Score durchschnittlich bei 2,27 ± 0,49.

**Table 1:** 

Einschlusskriterien	Ausschlusskriterien
Patienten mit primärer Gonarthrose und Indikation für eine Knie-TEP; auch Patienten mit bereits kontralateraler Knie-TEP	Sekundäre Gonarthrose (z. B. rheumatoide Arthritis, posttraumatische Arthrose), ligamentäre Instabilität, Achsfehlstellung (kindliche Fehlstellungen)
Vollständiger Datensatz zu den gewünschten Zielparametern	Hemiprothesen oder Prothesen mit Retropatellarersatz
Aufklärung und Einverständnis zur Studienteilnahme	Nicht einwilligungsfähige Patienten

## Präoperative Ausgangswerte und Beugefähigkeit zur Entlassung

Von 182 Patienten erreichten 73 (40 %) das Ziel von mindestens 90° Flexion. Die Beugefähigkeit lag in dieser Gruppe im Schnitt bei 91,2° ± 3,1° (90–110°) bei einem mittleren Alter von 65,7 ± 7,5 (38,6–87,5) Jahren. Dagegen konnten 109 Patienten (60 %) mit durchschnittlich 65,0 ± 8,9 (40,5–86,0) Jahren diese Vorgabe nicht erfüllen und hatten eine mittlere Flexion von 70,4° ± 13,4° (40–85°) zum Entlassungszeitpunkt (*p* < 0,001; Tab. 2).

**Table 2:** 

	Zeitpunkt: präoperativ			Zeitpunkt: Entlassung		
	< 90° bei Entlassung	> 90° bei Entlassung	*p*-Wert	< 90° bei Entlassung	> 90° bei Entlassung	*p*-Wert
Anzahl Patienten	109	73	–	109	73	–
männlich vs. weiblich	44 vs. 65	42 vs. 31	–	44 vs. 65	42 vs. 31	–
Flexion	120° ± 12°	121° ± 11°	0,68	70° ± 13°	91° ± 3°	*p* < 0,001
Streckdefizit	5° ± 6°	6° ± 6°	0,18	–	–	–
KSS Knee Score	52 ± 13	53 ± 13	0,74	–	–	–
KSS Function Score	56 ± 19	59 ± 18	0,19	–	–	–
KSS Total Score	108 ± 26	112 ± 26	0,27	–	–	–
WOMAC	46 ± 19	43 ± 21	0,33	–	–	–
SF-36	51 ± 15	53 ± 17	0,62	–	–	–
EQ-5D	8 ± 3	8 ± 3	0,8	–	–	–
VAS Schmerz	7 ± 2	6 ± 2	0,01	–	–	–

## Postoperatives Outcome nach 6 Wochen und nach einem Jahr

Im Beobachtungsverlauf konnte sich 6 Wochen postoperativ bei einer Differenz von 6,7° zwischen den beiden Gruppen noch ein signifikanter Unterschied in der ROM feststellen lassen (*p* = 0,001). Ein bestehendes Streckdefizit belief sich in der Differenz auf 1,2° (*p* = 0,05). Alle weiteren Vergleichsparameter waren zu diesem Zeitpunkt statistisch nicht signifikant unterschiedlich (Tab. 3).

**Table 3:** 

	Zeitpunkt: 6 Wochen postoperativ			Zeitpunkt: ein Jahr postoperativ		
	< 90° bei Entlassung	> 90° bei Entlassung	*p*-Wert	< 90° bei Entlassung	> 90° bei Entlassung	*p*-Wert
Anzahl Patienten	109	73	–	109	73	–
männlich vs. weiblich	44 vs. 65	42 vs. 31	–	44 vs. 65	42 vs. 31	–
Flexion	106° ± 14°	112° ± 13°	0,001	120° ± 10°	122° ± 10°	0,57
Streckdefizit	3° ± 4°	2° ± 3°	0,05	1° ± 2°	1° ± 2	0,87
KSS Knee Score	79 ± 14	83 ± 12	0,13	92 ± 8	90 ± 10	0,13
KSS Function Score	65 ± 19	69 ± 19	0,23	89 ± 12	90 ± 12	0,65
KSS Total Score	145 ± 28	151 ± 26	0,11	182 ± 18	179 ± 19	0,31
WOMAC	22 ± 12	21 ± 15	0,7	14 ± 12	15 ± 14	0,46
SF-36	61 ± 15	61 ± 16	0,87	75 ± 14	74 ± 17	0,53
EQ-5D	4 ± 3	4 ± 3	0,5	2 ± 2	2 ± 2	0,7
VAS Schmerz	3 ± 2	3 ± 2	0,36	2 ± 2	2 ± 2	0,38

Bis zur Jahreskontrolle näherte sich die Flexion der beiden Populationen in der ROM weiter an und es zeigten sich keine Abweichungen mehr. Auch alle anderen Scores bezüglich Schmerzen, Funktionalität und Lebensqualität waren ohne Unterschied zwischen den Gruppen (Tab. 3).

## Diskussion

Als Hauptergebnis der vorliegenden Studie zeigt sich, dass die Flexion zum Zeitpunkt der Entlassung keine Vorhersagekraft für die Flexion, das funktionelle Ergebnis und die Lebensqualität ein Jahr nach Implantation einer Knie-TEP hat. Obwohl zum Entlassungszeitpunkt eine Differenz mit durchschnittlich 21° und damit ein deutlicher, signifikanter Unterschied zwischen den Patienten mit mehr bzw. weniger als 90°-Flexion feststellbar ist, ist dieser nach 6 Wochen bereits auf 6° reduziert und nach einem Jahr nicht mehr nachweisbar.

Bisherige Literatur, die insbesondere die wissenschaftliche Basis der Qualitätskriterien des IQTiG bildete, ging davon aus, dass eine Flexion von mindestens 90° nötig ist, um Alltagaktivitäten wie Laufen oder Treppensteigen zufriedenstellend auszuüben [9, 15, 18].

In dieser Studie ist als weiteres Ergebnis sichtbar, dass sich auch die Lebensqualität der Patienten zwischen den Gruppen nicht unterscheidet. Ein Nichterreichen des 90°-Ziels zur Entlassung beeinflusst also nicht die Lebensqualität der Patienten. An dieser Stelle sollte allerdings auch erwähnt werden, dass die Mitarbeit des Patienten entscheidend für die Wiederherstellung seiner körperlichen Funktionen ist, sodass das Erreichen der 90° zumindest als Motivation für den Patienten genutzt werden kann [4, 13, 24].

Durch ein gewissenhaftes präoperatives Patientenmanagement kann die Komplikationsrate des Eingriffs möglichst geringgehalten werden. Unter diesen Bedingungen lag 2019 in Deutschland die Rate für allgemeine behandlungsbedürftige Komplikationen bei 2,4 % und für spezifische bei 1,6 % [10]. Ganz allgemein gilt für die totale Knieendoprothese ein 30-Tage-Mortalitätsrisiko der Operation von 0,08 % [6], ein Revisionsrisiko innerhalb der ersten 10 Jahre von 8 % [25] und eine Lebensdauer des Gelenksersatzes nach 25 Jahren von 82 % [3, 20].

Zur Diskussion sind schließlich noch einige Einflussfaktoren verblieben, die die Studienergebnisse potenziell beeinflusst haben könnten, insbesondere der individuelle Risikoscore. Auswertungen des dänischen Knieregisters konnten zeigen, dass weder individuelle Komorbiditäten noch der ASA-Status Einfluss auf die Qualität von Langzeitergebnissen haben. Selbst das Alter hat keine Relevanz. Die Untersuchung bezog sich hierbei auf Wiederaufnahme, Reoperation und 90-Tage-Mortalität und bildete damit die relevantesten Kriterien bezüglich der Patientengesundheit ab [5, 13]. Auch potenzielle Risikofaktoren, wie Diabetes Typ II, Alkoholabusus, Rauchen und ein BMI über 25 kg/m^2^, hatten keine Auswirkungen auf die postoperative Morbidität [8, 11, 12]. In der vorliegenden Untersuchung unterschieden sich die post hoc erstellten Gruppen nicht bezüglich Alter und ASA-Klassifikation.

Als letzter Einflussfaktor ist die Rehabilitation zu nennen, die, angefangen bei der Anästhesiemethode [7, 22] bis hin zur Frührehabilitation am Operationstag selbst, bessere Ergebnisse in den kurzzeitigen Funktionalitätsparametern, eine weitere Reduktion der LOS sowie einen niedrigeren Bedarf an Opioiden bringen kann [21]. Da die frühpostoperative Rehabilitation bei allen Patienten identisch war, kann diese als Confounder ausgeschossen werden. Auch die präoperative Beweglichkeit unterschied sich nicht zwischen den beiden Untersuchungsgruppen, sodass dieser stärkste Prädiktor der postoperativen Beweglichkeit ebenfalls als Confounder ausgeschlossen werden kann [19].

## Fazit für die Praxis

Zusammenfassend kann weder ein Vorteil noch ein Nachteil durch das Erreichen einer Flexion von 90° zum Entlassungszeitpunkt festgestellt werden. Auch ein vermindertes Bewegungsausmaß zur Entlassung, beispielsweise durch einen verkürzten stationären Aufenthalt, stellt für den Patienten keinen Nachteil dar. Für Flexion, Funktion und Lebensqualität ein Jahr nach der Operation ist die ROM zur Entlassung irrelevant. Damit ist nach den Ergebnissen dieser Studie das 90°-Kriterium nach Knietotalendoprothese kein adäquater Indikator für die mittelfristige Ergebnisqualität.
